# Implementation of the Amsterdam Pediatric Wrist Rules

**DOI:** 10.1007/s00247-018-4186-9

**Published:** 2018-07-10

**Authors:** Marjolein A. M. Mulders, Monique M. J. Walenkamp, Annelie Slaar, Frank Ouwehand, Nico L. Sosef, Romuald van Velde, J. Carel Goslings, Niels W. L. Schep

**Affiliations:** 1 Trauma Unit, Department of Surgery, Academic Medical Center, P.O. Box 22660, Meibergdreef, 91105 AZ Amsterdam, The Netherlands; 2 Department of Radiology, Westfriesgasthuis, P.O. Box 600, 1620 AR Hoorn, The Netherlands; 3 Emergency Department, Academic Medical Center, P.O. Box 22660, 1100 DD Amsterdam, The Netherlands; 4 Department of Surgery, Spaarne Gasthuis, P.O. Box 770, 2130 AT Hoofddorp, The Netherlands; 5 Department of Surgery, Tergooi Hospitals, P.O. Box 10016, 1201 DA Hilversum, The Netherlands; 6 Department of Surgery, Onze Lieve Vrouwe Gasthuis, P.O. Box 95500, 1090 HM Amsterdam, The Netherlands; 7 Department of Trauma and Hand Surgery, Maasstad Hospital, P.O. Box 9100, 3007 AC Rotterdam, The Netherlands

**Keywords:** Adolescents, Children, Decision rule, Distal forearm, Fracture, Radiography, Trauma, Wrist

## Abstract

**Background:**

The Amsterdam Pediatric Wrist Rules have been developed and validated to reduce wrist radiographs following wrist trauma in pediatric patients. However, the actual impact should be evaluated in an implementation study.

**Objective:**

To evaluate the effect of implementation of the Amsterdam Pediatric Wrist Rules at the emergency department.

**Materials and methods:**

A before-and-after comparative prospective cohort study was conducted, including all consecutive patients aged 3 to 18 years presenting at the emergency department with acute wrist trauma. The primary outcome was the difference in the number of wrist radiographs before and after implementation. Secondary outcomes were the number of clinically relevant missed fractures of the distal forearm, the difference in length of stay at the emergency department and physician compliance with the Amsterdam Pediatric Wrist Rules.

**Results:**

A total of 408 patients were included. The absolute reduction in radiographs was 19% compared to before implementation (chi-square test, *P*<0.001). Non-fracture patients who were discharged without a wrist radiograph had a 26-min shorter stay at the emergency department compared to patients who received a wrist radiograph (68 min vs. 94 min; Mann-Whitney *U* test, *P*=0.004). Eight fractures were missed following the recommendation of the Amsterdam Pediatric Wrist Rules. However, only four of them were clinically relevant.

**Conclusion:**

Implementing the Amsterdam Pediatric Wrist Rules resulted in a significant reduction in wrist radiographs and time spent at the emergency department. The Amsterdam Pediatric Wrist Rules were able to correctly identify 98% of all clinically relevant distal forearm fractures.

**Electronic supplementary material:**

The online version of this article (10.1007/s00247-018-4186-9) contains supplementary material, which is available to authorized users.

## Introduction

A wrist fracture is one of the most common fractures in children, accounting for 20–36% of all pediatric fractures [1–3]. The incidence of distal forearm fractures is increasing [4, 5], resulting in an increasing number of emergency department presentations and requested radiographs and, consequently, rising health care costs [6]. An important cause for the rise in health care costs is the increase in the number of requested radiographs [7].

Today, radiographic imaging in children with wrist trauma is performed routinely, even though only half of these patients sustained a wrist fracture [8]. To be more selective in the request for a radiograph and support physicians, the Amsterdam Pediatric Wrist Rules were developed and externally validated. Based on age and a number of clinical variables, the Amsterdam Pediatric Wrist Rules calculates the probability of a distal forearm fracture in children. The recommendation to obtain a radiograph is based on this probability. After external validation, the Amsterdam Pediatric Wrist Rules have been shown to have an acceptable sensitivity and a reduction in radiographs of 22% without missing any clinically relevant fractures [9]. The Amsterdam Pediatric Wrist Rules can potentially reduce the number of requested radiographs, reduce the length of stay at the emergency department, and thereby potentially reduce health care costs. However, the actual impact of decision rules, such as the Amsterdam Pediatric Wrist Rules, should be evaluated in an implementation study.

Therefore, the aim of this study was to evaluate the effect of implementation of the Amsterdam Pediatric Wrist Rules at the emergency department.

## Materials and methods

### Study design

This study was designed as a before-and-after comparative prospective cohort study. A prospective cohort of patients in which the Amsterdam Pediatric Wrist Rules were implemented (after group) was compared with a historical reference group in which the Amsterdam Pediatric Wrist Rules had been developed and validated (before group) [9]. Patients in the before and after groups were included in the same four hospitals to minimize disparity in patient characteristics and thus obtain comparable cohorts. Approval was obtained from the Ethics Committee and Institutional Review Board on October 8, 2014, and the board of directors of the participating centres. This study is registered with the Dutch Trial Register (NTR5105).

### Study population

In the after group, all consecutive patients aged 3 to 18 years presenting at the emergency department with an acute wrist trauma were included. Four hospitals were involved in recruiting patients: one academic and three teaching hospitals. An acute wrist trauma was defined as any high or low energetic accident involving the wrist, within 72 h preceding presentation at the emergency department. The wrist was defined as the proximal segment of the hand, including the distal part of the radius and ulna.

We excluded all patients who sustained a wrist injury more than 72 h before presentation at the emergency department, and patients who sustained multiple injuries with an Injury Severity Score (ISS) over 15. Patients whose radiograph were requested before their presentation at the emergency department (e.g., by their general practitioner), and patients with a previous fracture in the past 3 months were excluded as well. A log of patients who were screened for eligibility was kept for each participating centre.

**Fig. 1:**
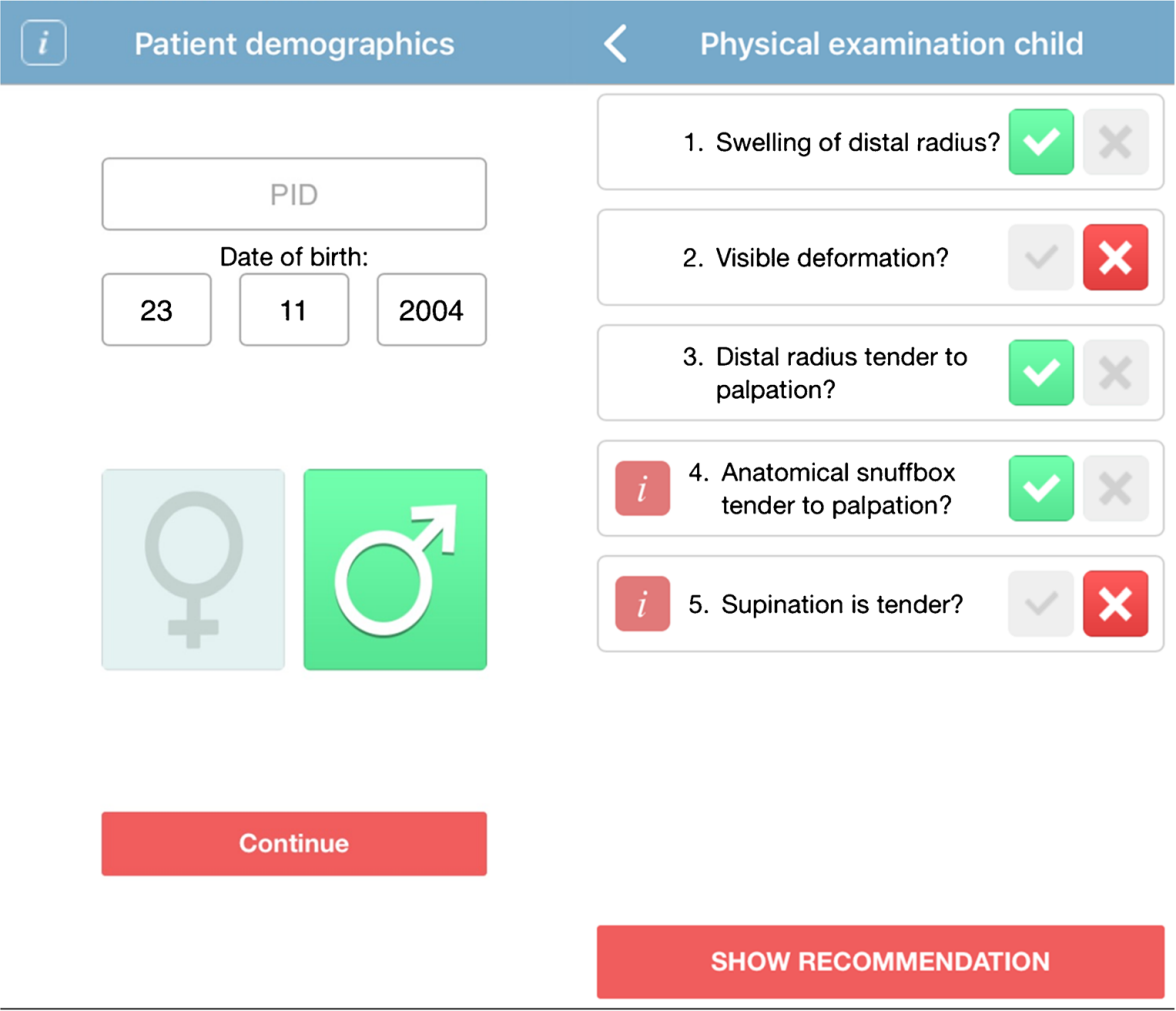
Amsterdam Pediatric Wrist Rules mobile application: patient demographics and clinical findings

**Fig. 2:**
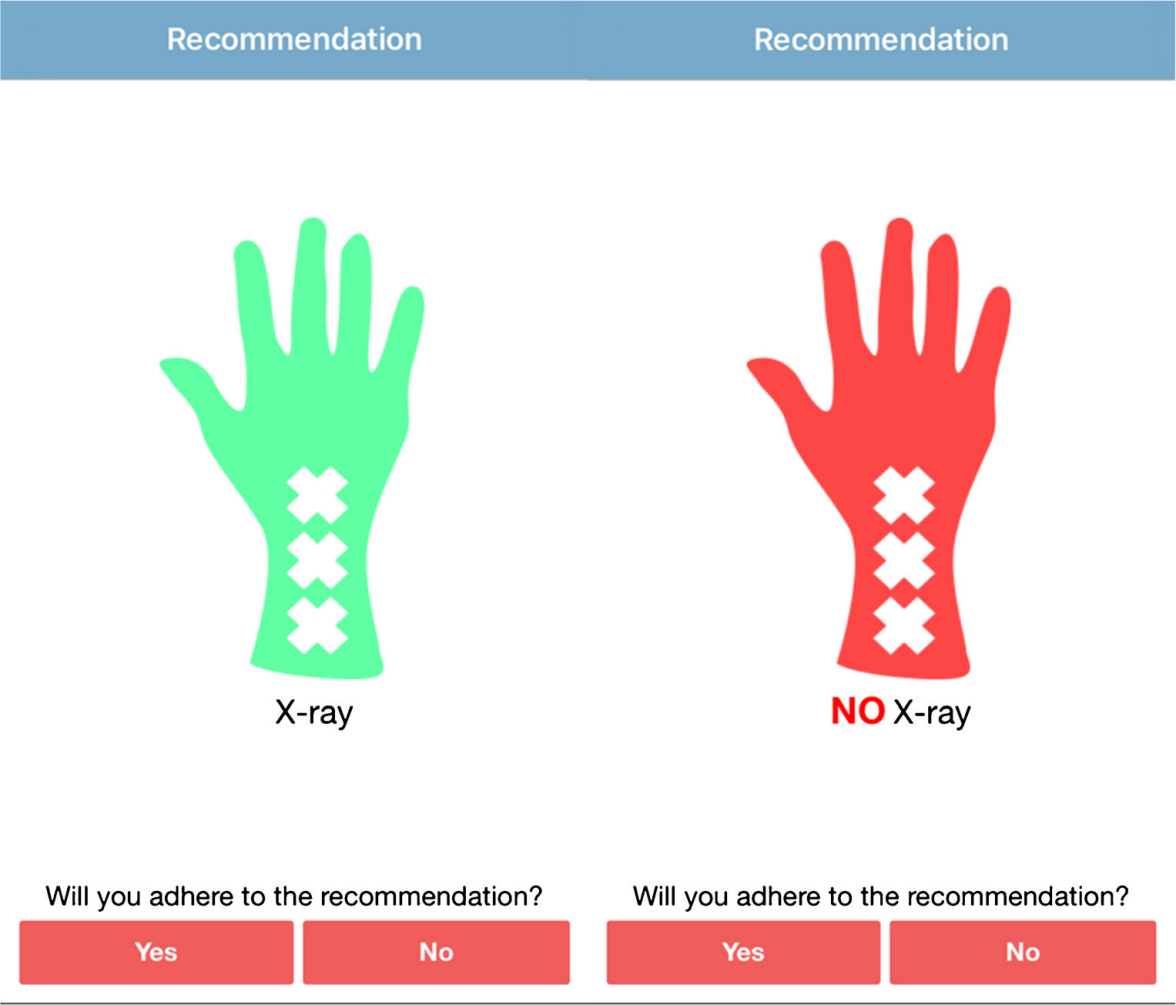
Amsterdam Pediatric Wrist Rules mobile application: recommendation wrist radiograph or not

Patients were included using the Amsterdam Pediatric Wrist Rules mobile application. The application is designed for both children and adults, and it distinguishes which decision rule to use based on the patient’s date of birth. First, the date of birth and sex were entered into the mobile application, as well as clinical findings during physical examination (Fig. 1). The clinical findings included swelling of the distal radius, visible deformation, painful palpation of the distal radius, painful palpation of the anatomical snuffbox and painful supination. Based on these findings, the Amsterdam Pediatric Wrist Rules calculates the probability of a distal forearm fracture. Based on this probability, a recommendation to obtain a wrist radiograph or not was given (Fig. 2). The application was also available as a calculator on the study website (www.amsterdamwristrules.nl).

The before group consisted of all patients aged 3 to 18 years between April 2011 and April 2014. The same inclusion and exclusion criteria were applied as in the after group. The Amsterdam Pediatric Wrist Rules were developed and validated in this historical reference group [9].

### Outcomes

The primary outcome was the difference in the number of wrist radiographs before and after implementation of the Amsterdam Pediatric Wrist Rules. A wrist radiograph was defined as a lateral and PA radiograph of the distal radius, ulna and the carpal bones.

Secondary outcomes were the number of clinically relevant missed fractures of the distal forearm, the difference in length of stay at the emergency department before and after implementation of the Amsterdam Pediatric Wrist Rules, physician compliance with the Amsterdam Pediatric Wrist Rules, and patient satisfaction with the received care at the emergency department. A fracture was defined as a disruption of one or more cortices of the radius or ulna. A fracture of both the distal radius and ulna, an antebrachial fracture, was recorded as one fracture. Fissures, avulsions of bony fragments and torus (or buckle) fractures were considered to be fractures as well. Carpal fractures were not taken into account since the incidence of carpal fractures in children is low and they are often occult on plain radiographs [4, 10, 11]. A clinically relevant fracture was defined as a fracture for which treatment or prognosis would have been affected by the missed or delayed radiographic diagnosis [12]. Therefore, a torus fracture was considered clinically irrelevant, as patients with a torus fracture can be safely treated with a bandage or removable splint [13–17]. In addition, we expected that if children have a lot of pain, physicians are more likely to apply a plaster for pain regulation. Fractures that received a plaster for pain regulation only were also considered clinically irrelevant. Fractures were judged to be clinically relevant independently by a radiologist and an orthopaedic trauma surgeon.

The length of stay at the emergency department was defined as the time between when patients presented and when they left the department. The difference in length of stay between the before and after groups was determined by comparing non-fracture patients with a wrist radiograph with non-fracture patients without a wrist radiograph due to the Amsterdam Pediatric Wrist Rules.

**Fig. 3:**
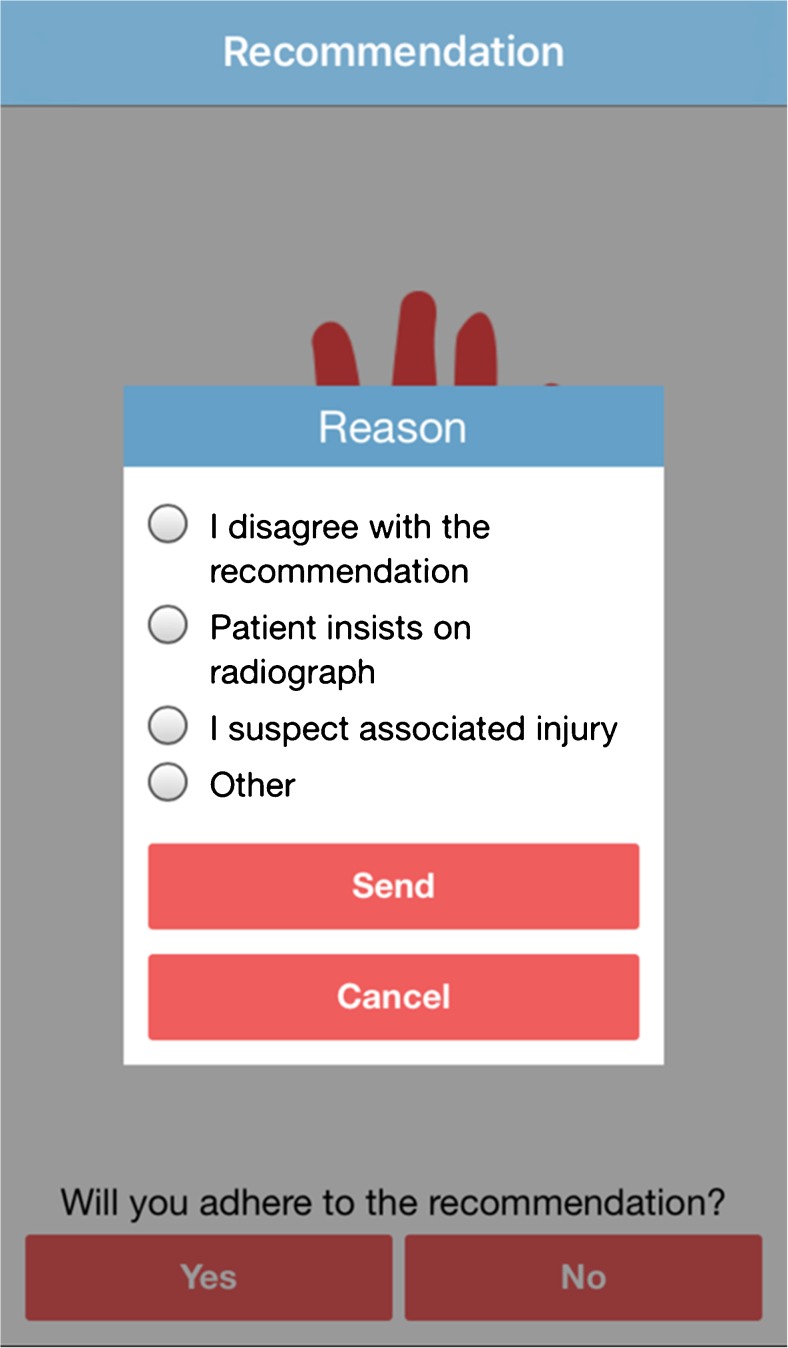
Amsterdam Pediatric Wrist Rules mobile application: physician compliance

Physician compliance with the Amsterdam Pediatric Wrist Rules was assessed with an additional question in the mobile application after the recommendation was provided. Physicians were asked via a yes-no question if they were planning to adhere to the recommendation. In case not, four possible answers could be given: (1) I disagree with the recommendation, (2) Patient insists on radiograph, (3) I have the suspicion on an associated injury, and (4) Other (Fig. 3).

Patients in whom no radiograph was obtained were contacted by phone after 7 to 10 days. If they failed to meet all of the following criteria, they were invited to the outpatient clinic: (1) pain has decreased, (2) ability to use wrist has improved, (3) able to lift more than 1 kg (e.g., a book or toys), (4) able to push open a door, (5) has returned to normal daily activities (e.g., school and social activities), and (6) has no plans to see a physician about wrist. At the outpatient clinic, referral for any additional (radiographic) work-up was at the discretion of the treating physician. In addition, patients were asked if they visited another physician (e.g., general practitioner, physiotherapist or other emergency department physician) regarding the wrist trauma, and if this physician obtained a wrist radiograph or gave additional treatment. Moreover, patients were asked if they were satisfied with the care they received at the emergency department, and if they felt secure without a wrist radiograph having been obtained. If not, they were asked if they would have felt more secure if a wrist radiograph had been made. Lastly, they were asked, in order to be 100% sure a fracture was ruled out, if they would have been willing to wait longer at the emergency department.

### Sample size and statistical analysis

The sample size calculation was based on our primary outcome: the difference in wrist radiographs before and after implementation of the Amsterdam Pediatric Wrist Rules. We assumed that before implementation, 90% of patients with a wrist trauma were sent for a wrist radiograph. We considered a minimal reduction in wrist radiographs of 9% to be feasible and relevant. Consequently, with an alpha of 5% and power of 90%, using the standard formula for superiority trials, 342 patients per group were required. Presuming a loss of 10% to follow-up, inclusion of at least 377 patients with a wrist trauma in whom the Amsterdam Pediatric Wrist Rules were applied was required. The same number of patients were required for the historical reference (before) group.

General descriptive statistics on baseline characteristics for both groups were performed. Differences in gender and fracture characteristics between the before and after groups were compared using a chi-square test. The difference in age between both groups was analysed using a Mann-Whitney *U* test. The same applied for differences in patient characteristics between the included patients and patients eligible for inclusion but not included. The primary outcome, the number of patients referred for a wrist radiograph before and after implementation, was compared using a chi-square test. Secondary outcomes were analysed using either a chi-square test for categorical data, and a Mann-Whitney *U* test for continuous data.

## Results

### Study participants

**Fig. 4:**
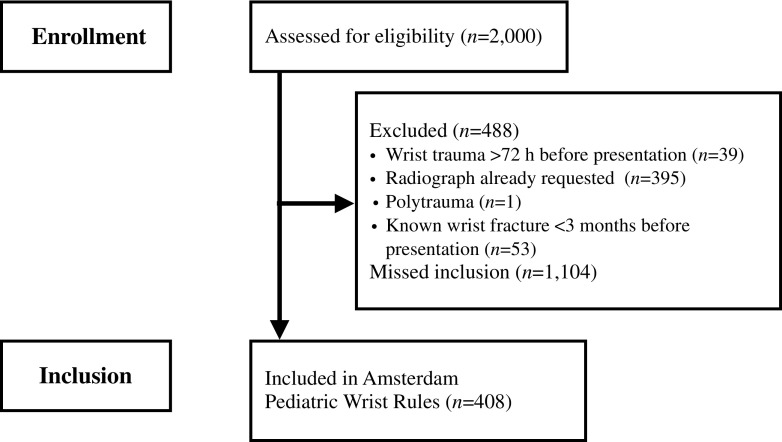
Flow diagram of patient selection

From November 2015 to June 2016, 408 patients were included. The majority of these patients were included at the teaching hospitals (84%). Since the registration of excluded patients was incomplete in the three teaching hospitals, a chart review was performed in these hospitals to complete the number of excluded patients and patients eligible but not included. A total of 2,000 patients were screened for eligibility, of which 488 patients were excluded and 1,104 patients were eligible but were not included (Fig. 4). Baseline characteristics between the included patients and patients eligible but not included were well balanced, except for age (Table 1).

**Table 1:** Baseline characteristics of included patients versus eligible but non-included patients

	Included in Amsterdam Pediatric Wrist Rules *n*=408	Eligible but non-included *n*=1,104	*P*-value
Age, median (interquartile range), years^a^	12 (9–14)	11 (7–13)	<0.001
Male, *n* (%)^b^	211 (51.7)	605 (54.8)	0.285
Distal forearm fracture, *n* (%)^b^	181 (44.4)	461 (41.8)	0.363
Distal radius fracture, *n* (%)	38 (78.5)	369 (80.0)	0.788
Distal ulna fracture, *n* (%)	1 (0.5)	2 (0.5)	0.788
Distal radius and ulna fractures, *n* (%)	142 (21.0)	90 (19.5)	0.788

^a^Mann-Whitney U test

^b^Chi-Square test

The median age of the 408 patients included after implementation was 12 years (interquartile range [IQR] 9–14 years), and 52% were male. Of all patients, 45% sustained a distal forearm fracture, of which 79% were distal radius fractures. The historical reference (before) group consisted of 799 patients. Baseline characteristics between the before and after groups were comparable, except for gender (the before group had significantly more males). Although the proportion of patients with a distal forearm fracture was not statistically different, more patients in the after group had a fracture of both the distal radius and ulna (Table 2).

**Table 2:** Baseline characteristics before and after implementation of the Amsterdam Pediatric Wrist Rules

	Before implementation of the Amsterdam Pediatric Wrist Rules *n*=799	After implementation of the Amsterdam Pediatric Wrist Rules *n*=408	*P*-value
Age, median (interquartile range), years^a^	11 (10–14)	12 (9–14)	0.490
Male, *n* (%)^b^	476 (59.6)	211 (51.7)	0.009
Distal forearm fracture, *n* (%)^b^	365 (45.7)	180 (44.1)	0.605
Distal radius fracture, *n* (%)	320 (87.7)	141 (78.3)	0.011
Distal ulna fracture, *n* (%)	3 (0.8)	1 (0.6)	0.011
Distal radius and ulna fractures, *n* (%)	42 (11.5)	38 (21.1)	0.011

^a^Mann-Whitney U test

^b^Chi-square test

### Reduction in radiographs and clinically relevant missed fractures

The absolute reduction in radiographs was 19% (99% vs. 80%; chi-square test, *P*<0.001) (Table 3). Eight fractures were missed following the recommendation of the Amsterdam Pediatric Wrist Rules (4.4%, Table 4). Only one of these patients received no wrist radiograph and this patient was contacted by phone after a week. The 12-year-old boy still complained about his wrist and he was invited to the outpatient clinic. A radiograph of the wrist was performed, which showed a Salter-Harris type II fracture (Online Resource 1). The patient was treated with a below-elbow plaster for 3 weeks, after which he had no residual complaints. This fracture was considered clinically relevant, since it was treated with a below-elbow plaster for 3 weeks. Among the seven other fractures, three fractures were judged clinically relevant (1.7%). Two fractures were epiphysiolysis fractures of the distal radius (Salter-Harris type II), which were both treated with a below-elbow plaster (Online Resources 2 and 3). One of these fractures, which occurred in a 10-year-old girl, was displaced and underwent closed reduction (Online Resource 3). The third clinically relevant fracture was a greenstick fracture with 8° of dorsal angulation (Online Resource 4). That 13-year-old boy was treated with a below-elbow plaster for 3 weeks. During immobilization, the angulation did not deteriorate. In all seven patients, the physicians had ignored the recommendation of the Amsterdam Pediatric Wrist Rules, which was to not perform a wrist radiograph. After implementation, the Amsterdam Pediatric Wrist Rules correctly identified 98% of all clinically relevant fractures (Table 5).

**Table 3:** Primary and secondary outcomes

	Before implementation of the Amsterdam Pediatric Wrist Rules *n*=799	After implementation of the Amsterdam Pediatric Wrist Rules *n*=408	*P*-value
Wrist radiographs, *n* (%)^a^	788 (98.6)	326 (79.9)	<0.001
Length of stay at the emergency department^b^, median (interquartile range), hours:minutes	1:41 (1:13–2:18)	1:41 (1:08–2:21)	0.493

^a^Mann-Whitney U test

^b^Chi-square test

**Table 4:** Missed fractures

Patient	Gender, age	Fracture type	Treatment	Clinically relevant
1	Male, 13 years	Greenstick fracture with 8 degrees dorsal angulation (see also Online resource 4)	Below-elbow plaster for 4 weeks	Yes, since clinical and radiologic follow-up was required to check if the angulation did not deteriorate
2	Female, 10 years	SH type II fracture with volar angulation (see also Online Resource 3)	Closed reduction and below-elbow plaster	Yes, since clinical and radiologic follow-up was required to check if the angulation did not deteriorate
3	Male, 12 years	Torus fracture	Below-elbow plaster	No, could be treated with a bandage or removable splint, or plaster for pain regulation only
4	Male, 12 years	Fractures type II fracture (see also Online Resource 1)	Below-elbow plaster	Yes, since it was treated with a below-elbow plaster
5	Male, 17 years	USP ulnar styloid process	Below-elbow plaster	No, could be treated with a bandage or removable splint, or plaster for pain regulation only
6	Male, 13 years	SH type II fracture	Below-elbow plaster	No, could be treated with a bandage or removable splint, or plaster for pain regulation only
7	Male, 13 years	Torus both-bone fracture	Below-elbow plaster	No, could be treated with a bandage or removable splint, or plaster for pain regulation only
8	Male, 16 years	SH type II fracture (see also Online Resource 2)	Below-elbow plaster	Yes, since clinical and radiologic follow-up was required to check if the angulation did not deteriorate

*SH* Salter-Harris

**Table 5:** Performance of the Amsterdam Pediatric Wrist Rules after implementation among 408 patients with wrist trauma

	No distal forearm fracture	Distal forearm fracture
Amsterdam Pediatric Wrist Rules recommends radiograph	155	172
Amsterdam Pediatric Wrist Rules recommends no radiograph	77	4
Sensitivity, % (95% confidence interval)	97.7% (93.9–99.3)	
Specificity, % (95% confidence interval)	33.2% (27.2–39.7)	

Performance was tested based on the 18.7% reduction in wrist radiographs, applying the definition of a clinically relevant fracture

### Length of stay at the emergency department

The length of stay at the emergency department before implementation was equal to the length of stay after implementation (Table 3). However, among the 228 non-fracture patients, patients who were discharged without a wrist radiograph due to the Amsterdam Pediatric Wrist Rules had a significant 26-min shorter stay compared to patients who had a wrist radiograph (68 min [IQR 39–97] vs. 94 min [IQR 64–136]; Mann-Whitney *U* test, *P*=0.004).

### Physician compliance

Both surgical residents and emergency physicians were involved in including patients at the emergency department. The Amsterdam Pediatric Wrist Rules recommended no wrist radiograph in 82 patients. In 31% of the patients, the physicians adhered to this recommendation. The main reason not to adhere to the recommendation was the suspicion of an associated injury (40%). Other reasons for not adhering to the recommendation were that the patient or one of the parents insisted on a radiograph (21%), the physician disagreed on the recommendation (14%), or another unspecified reason (25%).

Among the patients who received a wrist radiograph, despite the recommendation of the Amsterdam Pediatric Wrist Rules, there were seven patients who had sustained a distal forearm fracture. Furthermore, one patient had a fracture of both the fourth and the fifth metacarpal bones. Lastly, 15 patients were treated with a plaster cast due to a clinically suspected scaphoid fracture. None of these patients had a scaphoid fracture during reassessment at the outpatient clinic after a week.

Three radiographs of the hand were requested in patients who received no wrist radiograph. Two of them revealed no fracture. One of them showed a fracture of the fifth metacarpal bone.

### Additional consultations and patient satisfaction

Besides the patient who had complaints after a week and was sent to the outpatient clinic, only one patient visited a general practitioner. However, the general practitioner decided not to perform a radiograph and the patient received no additional treatment.

All patients were satisfied with their visit to the emergency department. Furthermore, besides the patient who still had complaints after 1 week, all patients felt secure about the fact that they did not receive a wrist radiograph. In addition, they would not have been willing to wait longer at the emergency department to be 100% sure a fracture of the wrist was ruled out.

## Discussion

Implementation of the Amsterdam Pediatric Wrist Rules could potentially result in an 19% reduction in wrist radiographs, and a 26-min reduction in time spent at the emergency department for non-fracture patients who had no wrist radiograph. The Amsterdam Pediatric Wrist Rules were able to correctly identify 98% of all clinically relevant distal forearm fractures.

The potential reduction in wrist radiographs was lower compared to what we expected based on the external validation study (19% vs. 22%). However, the sensitivity of the Amsterdam Pediatric Wrist Rules was 98%, which was higher than in the external validation (95.9%), meaning that the Amsterdam Pediatric Wrist Rules would correctly identify 98% of all clinically relevant distal forearm fractures. The negative predictive value was 95%, indicating that of all patients in whom the Amsterdam Pediatric Wrist Rules recommended not to make a radiograph, 95% did not have a clinically relevant fracture. However, four clinically relevant fractures were missed following the recommendation of the Amsterdam Pediatric Wrist Rules. In three of these patients, the physicians did not adhere to the recommendation. This highlights an important point, since the Amsterdam Pediatric Wrist Rules are not designed to replace the clinical experience and judgment of the physician. Rather, they were developed as a validated tool to guide physicians in deciding whether to request a wrist radiograph in children suspected of a distal forearm fracture. However, if there is a suspected associated injury (e.g., a fracture of one of the carpal bones or a metacarpal fracture), appropriate radiographs should be performed. In addition, patients and their parents should be adequately counselled and advised to contact their general practitioner if complaints persist or do not diminish. During our telephone survey, all patients felt secure about the fact that they did not receive a wrist radiograph. The patients or their parents indicated that a wrist radiograph would not be necessary if proper counselling would be given. In addition, they would not have been willing to wait longer at the emergency department to be 100% sure a wrist fracture was ruled out. Counselling a patient will take only a few minutes, while conducting and reviewing a radiograph, and explaining the findings to the patient takes more time. This was confirmed by the significant reduction of 26 min in time spent at the emergency department for non-fracture patients in whom no radiograph was performed compared to non-fracture patients who had a wrist radiograph.

To decrease the number of clinically relevant missed fractures of the distal forearm, it would be possible to lower the threshold of the predicted probability for the recommendation. This threshold was set at 23%, and was based on the available literature considering the fact that three avoided radiographs would outweigh one missed fracture [9, 18]. If we would decrease this threshold to, for example 15%, we would achieve a higher sensitivity (99%). However, we would still miss one clinically relevant fracture. Furthermore, the reduction in radiographs would decrease to 12%. The reduction in radiographs, together with the reduction in time spent at the emergency department, could result in potential cost savings. Therefore, we are currently undertaking a cost analysis and budget impact analysis, taking into account direct medical costs (e.g., costs of wrist radiograph, costs for consultation at the emergency department and appointment at the outpatient clinic) and indirect medical costs (e.g., reduction in time spent at the emergency department). In addition, due to the reduction in radiographs and time spent at the emergency department, other patients could potentially benefit due to a better use of resources.

The Amsterdam Pediatric Wrist Rules were used by a variety of different physicians. However, only 31% of the physicians adhered to the recommendation of the rules, resulting in a 6% reduction in radiographs requested. Although the reduction was lower than the potential reduction of 19%, it was still a statistically significant difference compared to before implementation. The mean reason for not adhering to the recommendation was the suspicion of an associated injury. However, only two patients had a fracture of the fourth and fifth metacarpal bones. Furthermore, of the 15 patients who received a wrist radiograph due to suspicion of a scaphoid fracture, none of them had a scaphoid fracture upon re-evaluation at the outpatient clinic after at least a week of plaster immobilization. Carpal fractures in children are uncommon injuries [1, 10]. In our cohort of patients, there were only two patients with a scaphoid and triquetrum fracture, respectively. Unfortunately, we are not able to determine the 25% unspecified reasons for not adhering to the recommendation of the Amsterdam Pediatric Wrist Rules. Despite the high rate of noncompliance, we expect that by showing that the Amsterdam Pediatric Wrist Rules can safely be used, the compliance will increase.

This study has some limitations. We chose a before-and-after comparative prospective design instead of a randomized study. The rationale behind this is that randomizing patients with wrist trauma is not feasible since the incidence is high and cognitive guidelines have been learned by the physicians [19]. Moreover, because of the high incidence of children with distal forearm trauma, randomizing would take a lot of time and put a lot of pressure on the emergency department. Since not randomizing patients can introduce selection bias, we tried to diminish this by aiming to include all consecutive children aged 3 to 18 years with wrist trauma. Although eligible but non-included patients were statistically younger, this difference in age was only 1 year (12 vs. 11 years). The percentage of patients eligible but not included was relatively high. This was due to the change in workflow at the emergency departments. When patients visit the emergency department, they are first seen by a triage nurse before they are either sent to the general practitioner or sent for a radiograph before an emergency physician examines the patient. Therefore, the next step will be to implement the Amsterdam Pediatric Wrist Rules by the emergency department triage nurse, which previously has been done for the Ottawa Ankle Rules and the Canadian C-spine Rule [20, 21].

Several attempts have been made to develop clinical decision rules for children with wrist trauma [22–24]. However, these studies were limited by small sample sizes and only the study by Webster et al. showed an acceptable sensitivity after external validation (99%) [25]. Yet, the specificity was low, resulting in a potential reduction in requested radiographs of only 7%. Therefore, it is questionable if this decision rule is a supplement to current practice. Although this reduction in radiographs is comparable with the actual reduction of the Amsterdam Pediatric Wrist Rules of 6%, the Amsterdam Pediatric Wrist Rules have a sensitivity of 98% after implementation, and a potential reduction in radiographs of 19%.

## Conclusion

The Amsterdam Pediatric Wrist Rules are the first validated and implemented clinical decision rules in children with a suspected fracture of the distal forearm. Implementation showed that the use of the Amsterdam Pediatric Wrist Rules results in a reduction in radiographs requested and time spent at the emergency department. Although the Amsterdam Pediatric Wrist Rules could correctly identify 98% of all clinically relevant distal forearm fractures, the clinical judgment and experience of the physician still play an important part in the decision-making process for a radiographic referral in children with a trauma of the wrist.

## Electronic supplementary material

Online Resource 1 A 12-year-old boy with a Salter-Harris type II fracture of the distal radius, considered clinically relevant (PNG 1633 kb)

High Resolution Image (TIF 245 kb)

Online Resource 2 A 16-year-old boy with a Salter-Harris type II fracture, considered clinically relevant (PNG 2004 kb)

High Resolution Image (TIF 275 kb)

Online Resource 3 A 10-year-old girl with a Salter-Harris type II distal radius fracture with volar angulation (**a**), with an acceptable closed reduction (**b**), considered clinically relevant (PNG 2055 kb)

High Resolution Image (TIF 281 kb)

DUMMY (PNG 2442 kb)

High Resolution Image (TIF 336 kb)

Online Resource 4 A 13-year-old boy with a greenstick fracture with 8° of dorsal angulation, considered clinically relevant (PNG 1373 kb)

High Resolution Image (TIF 1017 kb)
