# Supplementary figures and images for: Implementation of the Amsterdam Pediatric Wrist Rules
Source: Pediatr Radiol. 2018 Jul 10;48(11):1612–20. doi: 10.1007/s00247-018-4186-9 (PMC6153883; doi:10.1007/s00247-018-4186-9)

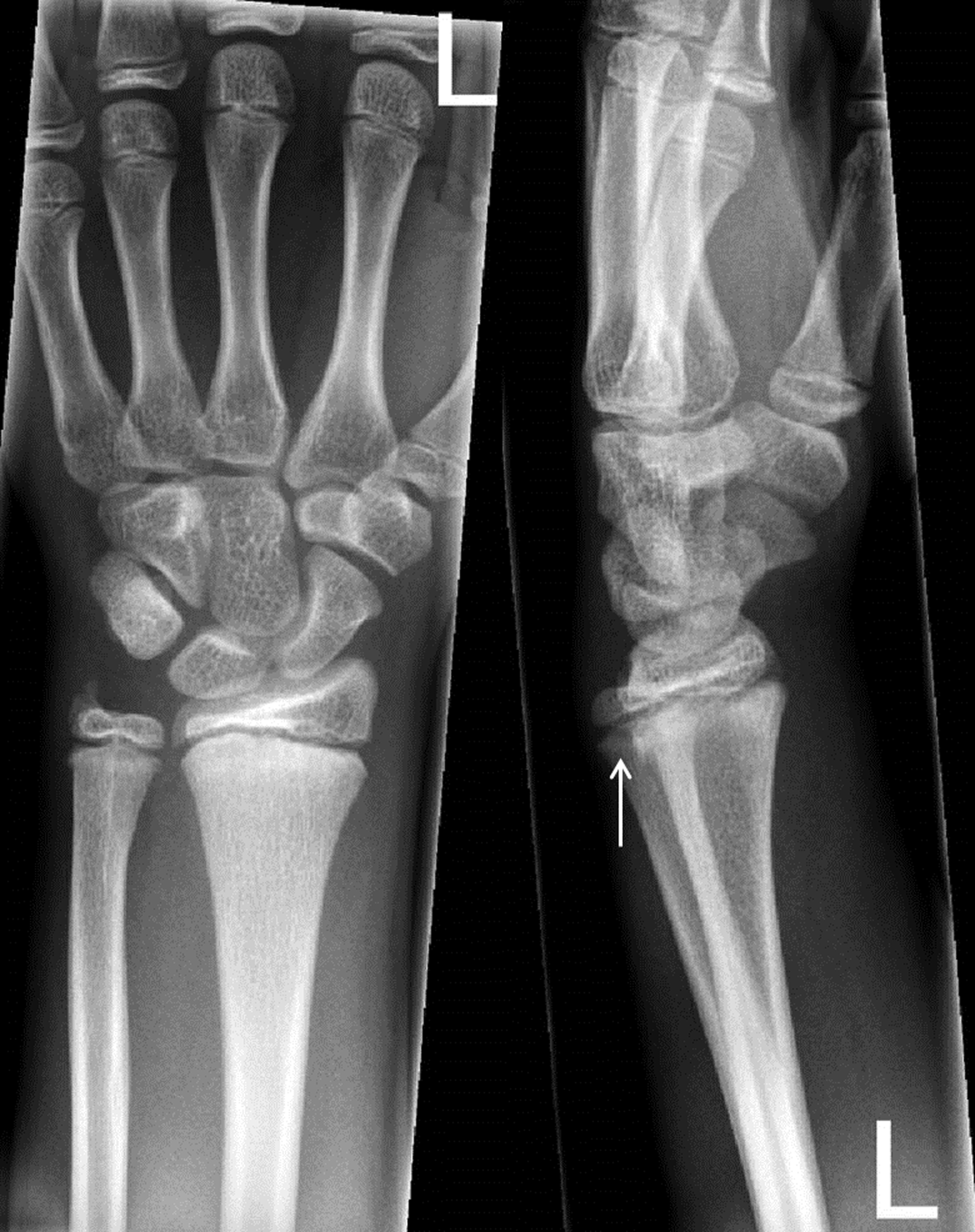

Supplement: Supplementary file 1 — A 12-year-old boy with a Salter-Harris type II fracture of the distal radius, considered clinically relevant (PNG 1633 kb) [file 247_2018_4186_Fig5_ESM.png]

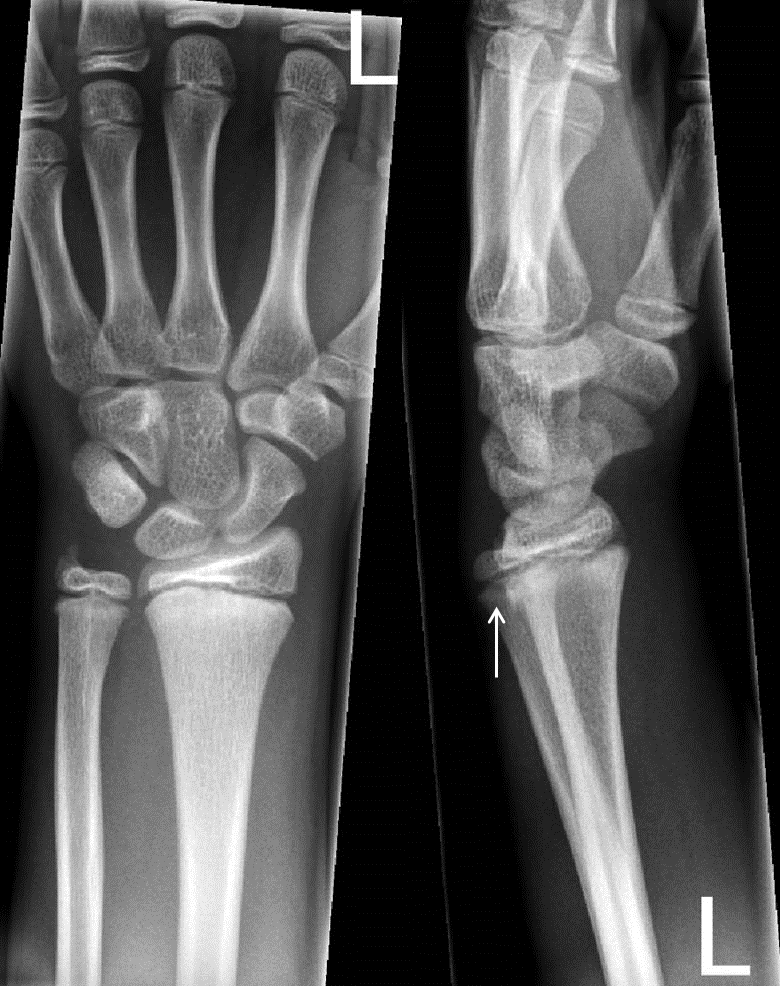

Supplement: Supplementary file 2 — High Resolution Image (TIF 245 kb) [file 247_2018_4186_MOESM1_ESM.tif]

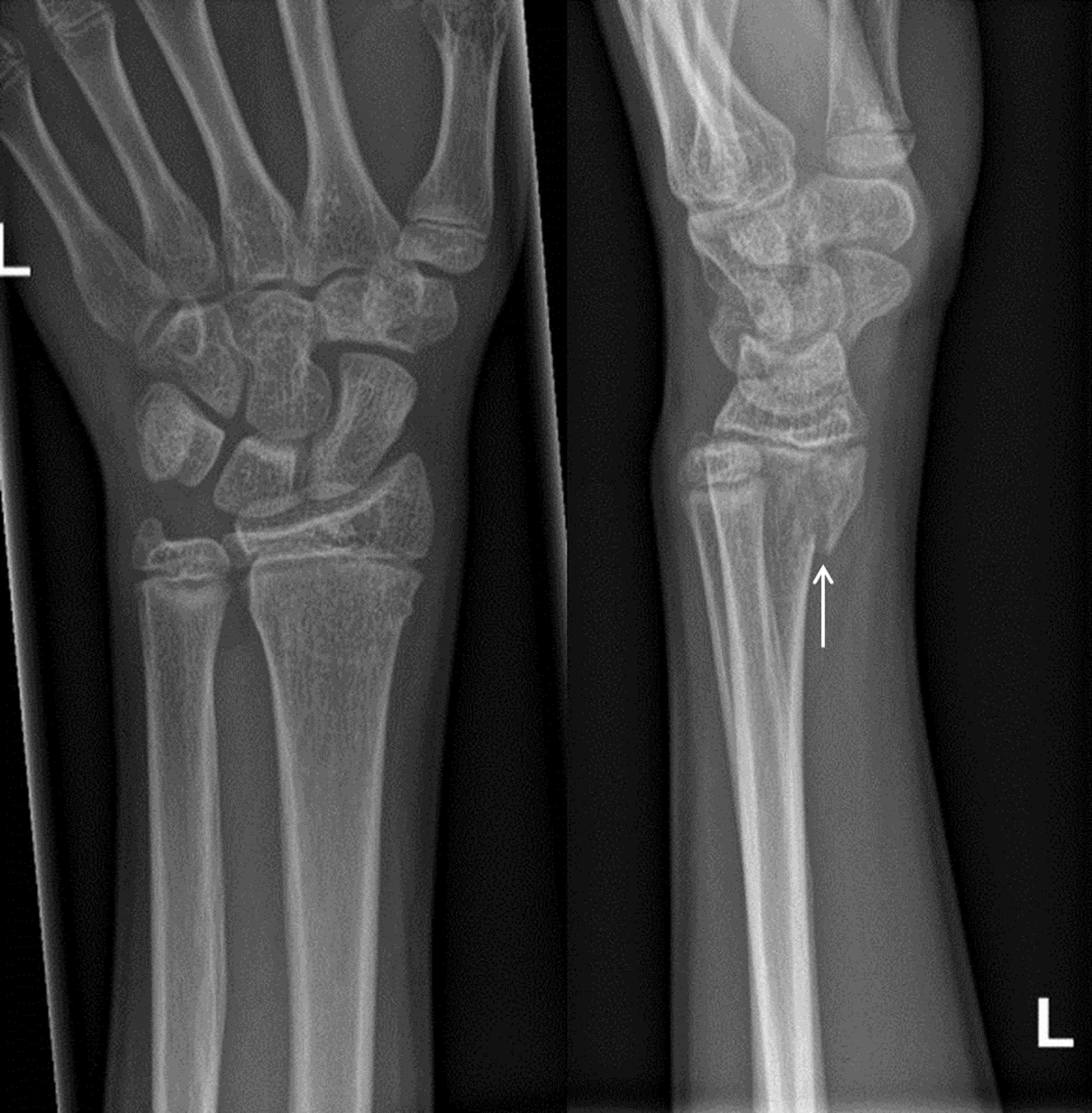

Supplement: Supplementary file 3 — A 16-year-old boy with a Salter-Harris type II fracture, considered clinically relevant (PNG 2004 kb) [file 247_2018_4186_Fig6_ESM.png]

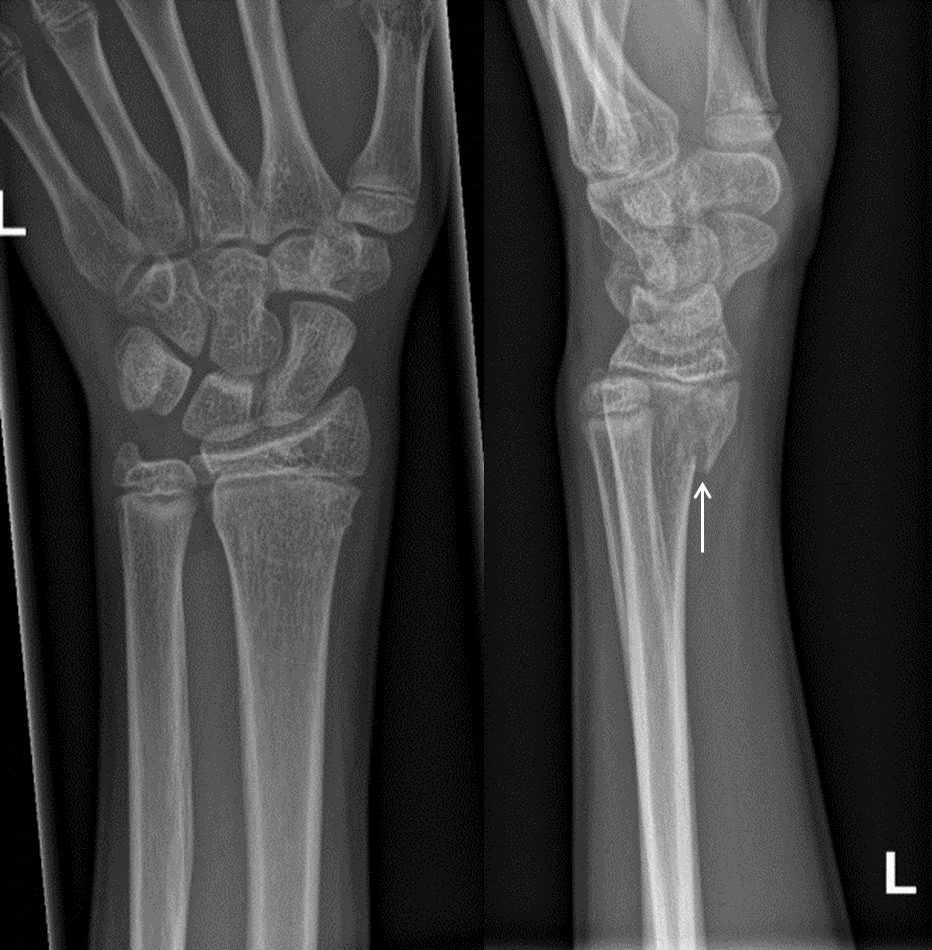

Supplement: Supplementary file 4 — High Resolution Image (TIF 275 kb) [file 247_2018_4186_MOESM2_ESM.tif]

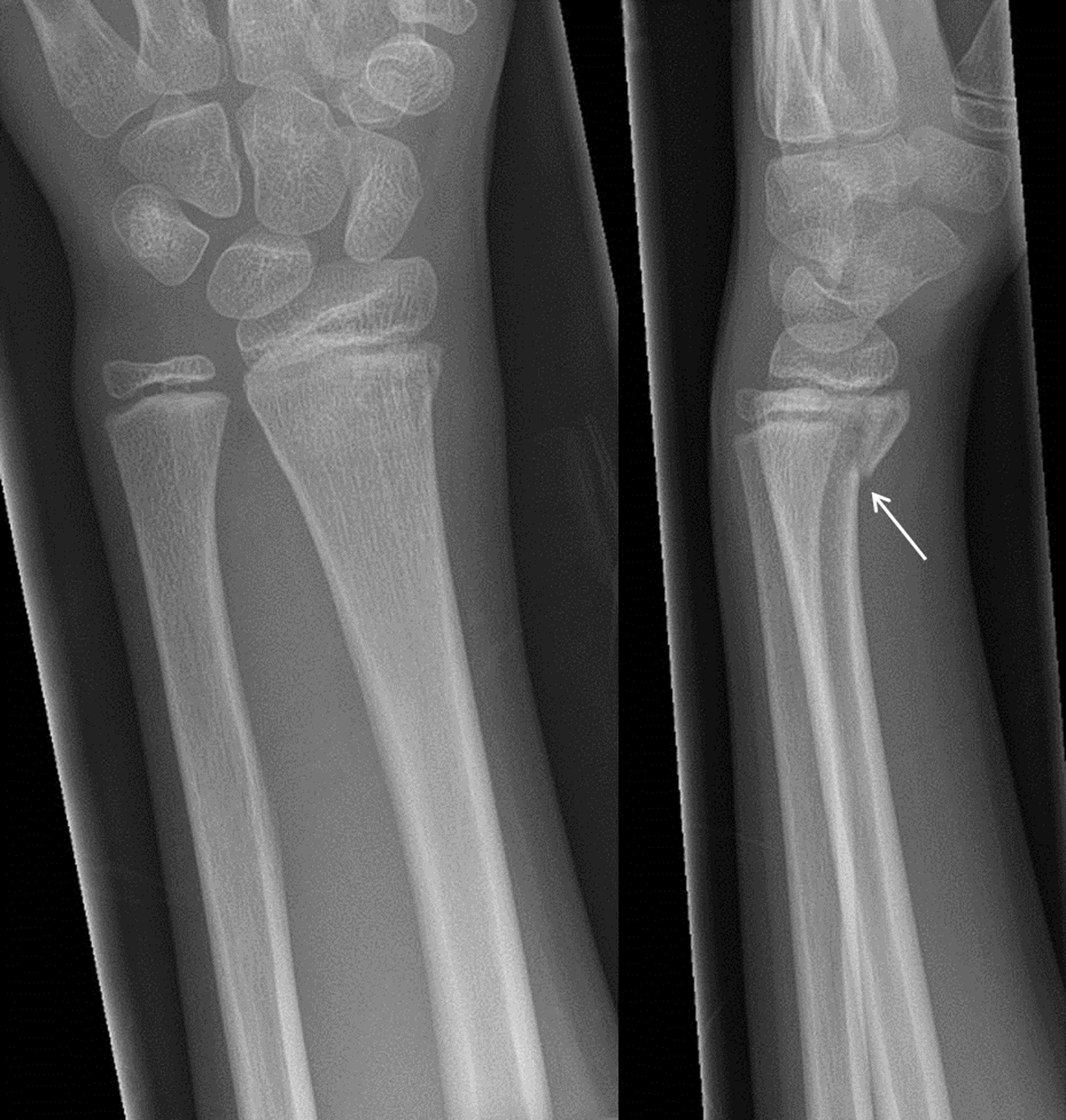

Supplement: Supplementary file 5 — A 10-year-old girl with a Salter-Harris type II distal radius fracture with volar angulation (a), with an acceptable closed reduction (b), considered clinically relevant (PNG 2055 kb) [file 247_2018_4186_Fig7_ESM.png]

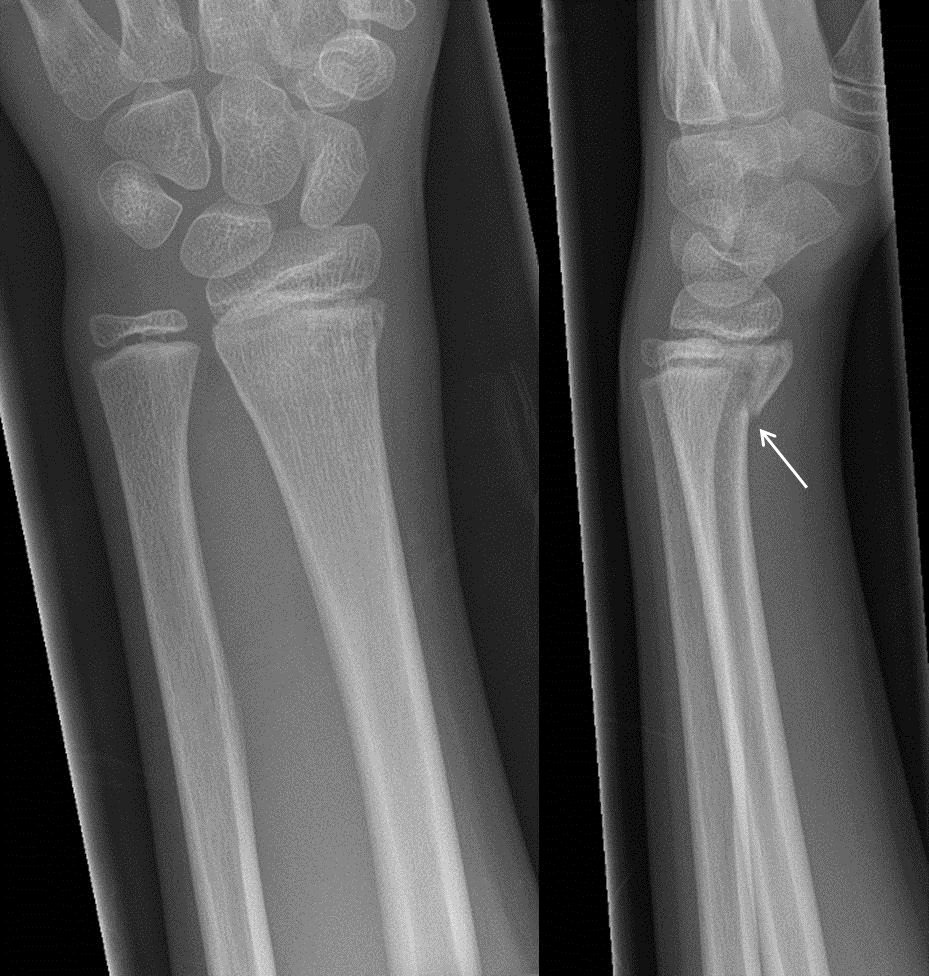

Supplement: Supplementary file 6 — High Resolution Image (TIF 281 kb) [file 247_2018_4186_MOESM3_ESM.tif]

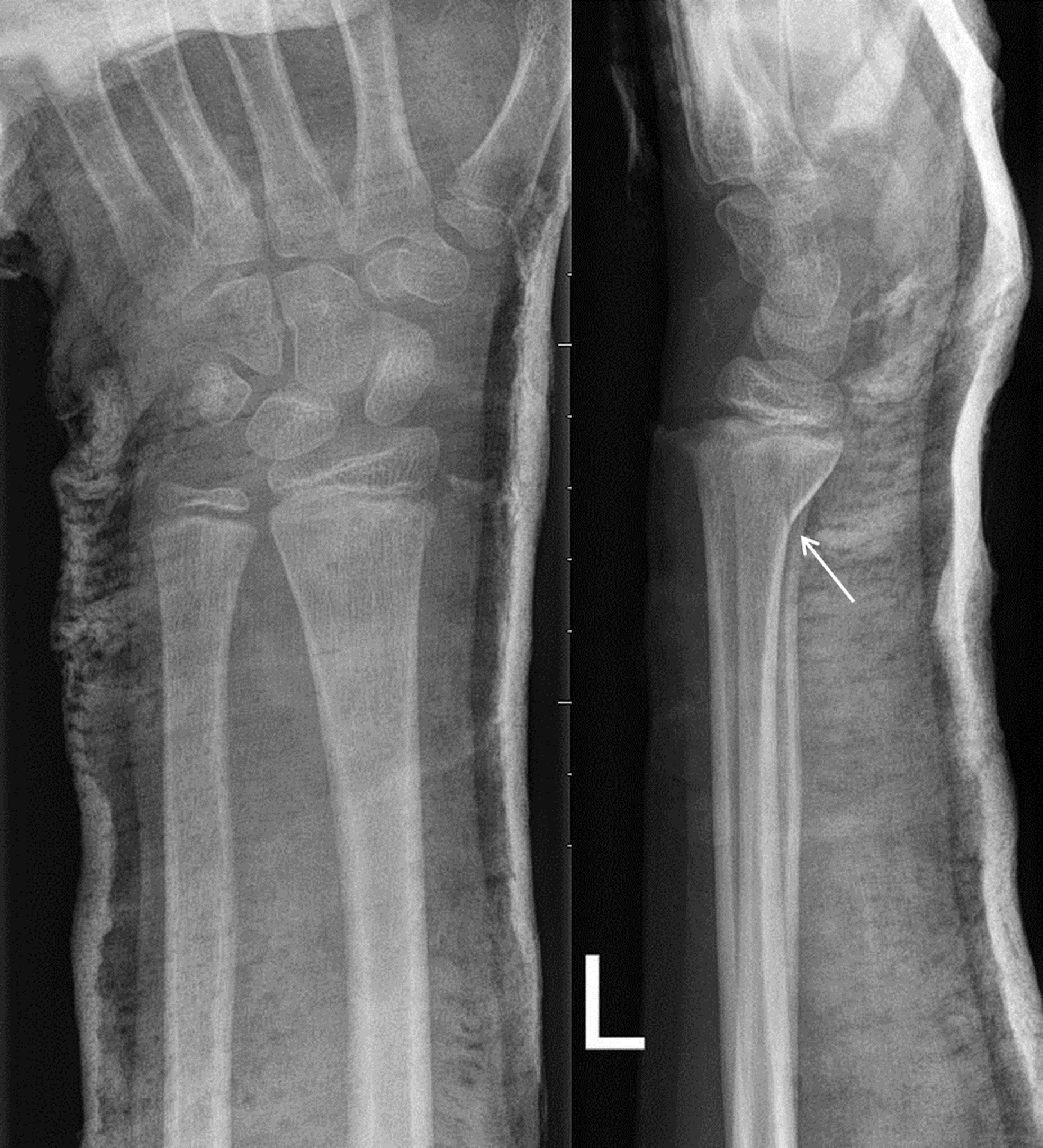

Supplement: Supplementary file 7 — (PNG 2442 kb) [file 247_2018_4186_Fig8_ESM.png]

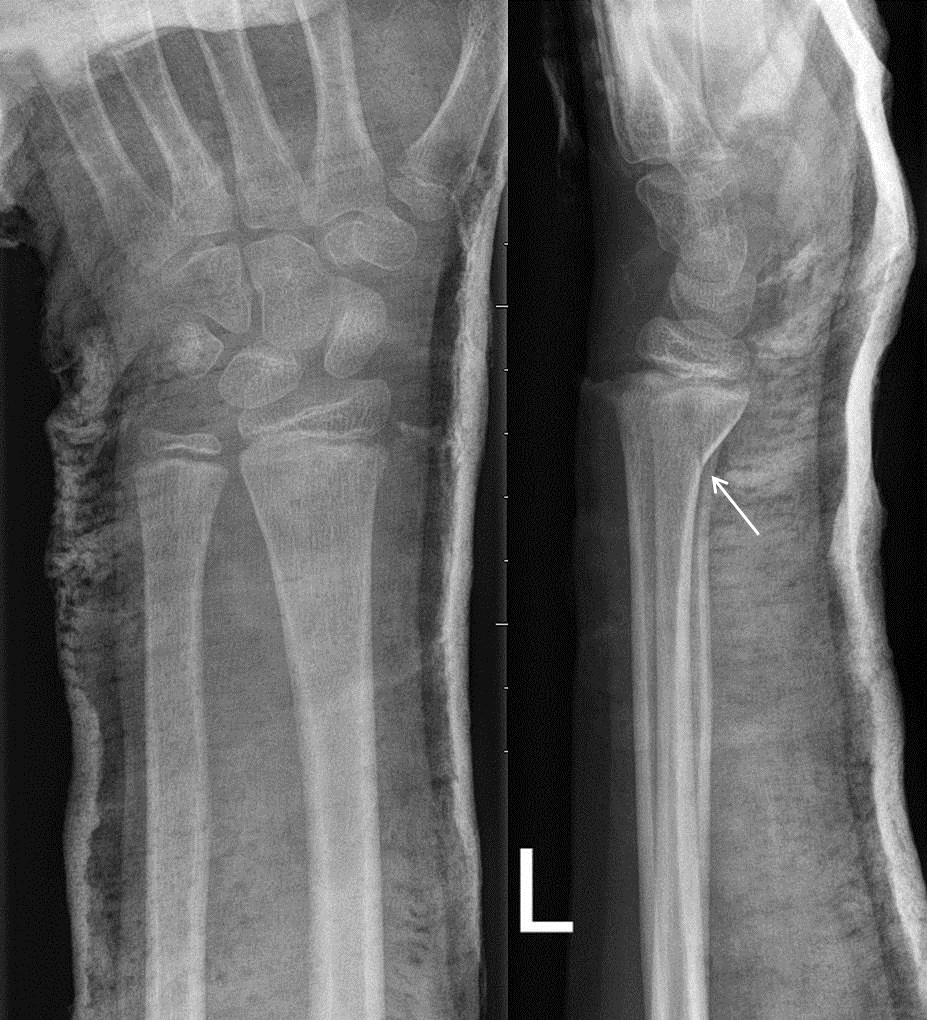

Supplement: Supplementary file 8 — High Resolution Image (TIF 336 kb) [file 247_2018_4186_MOESM4_ESM.tif]

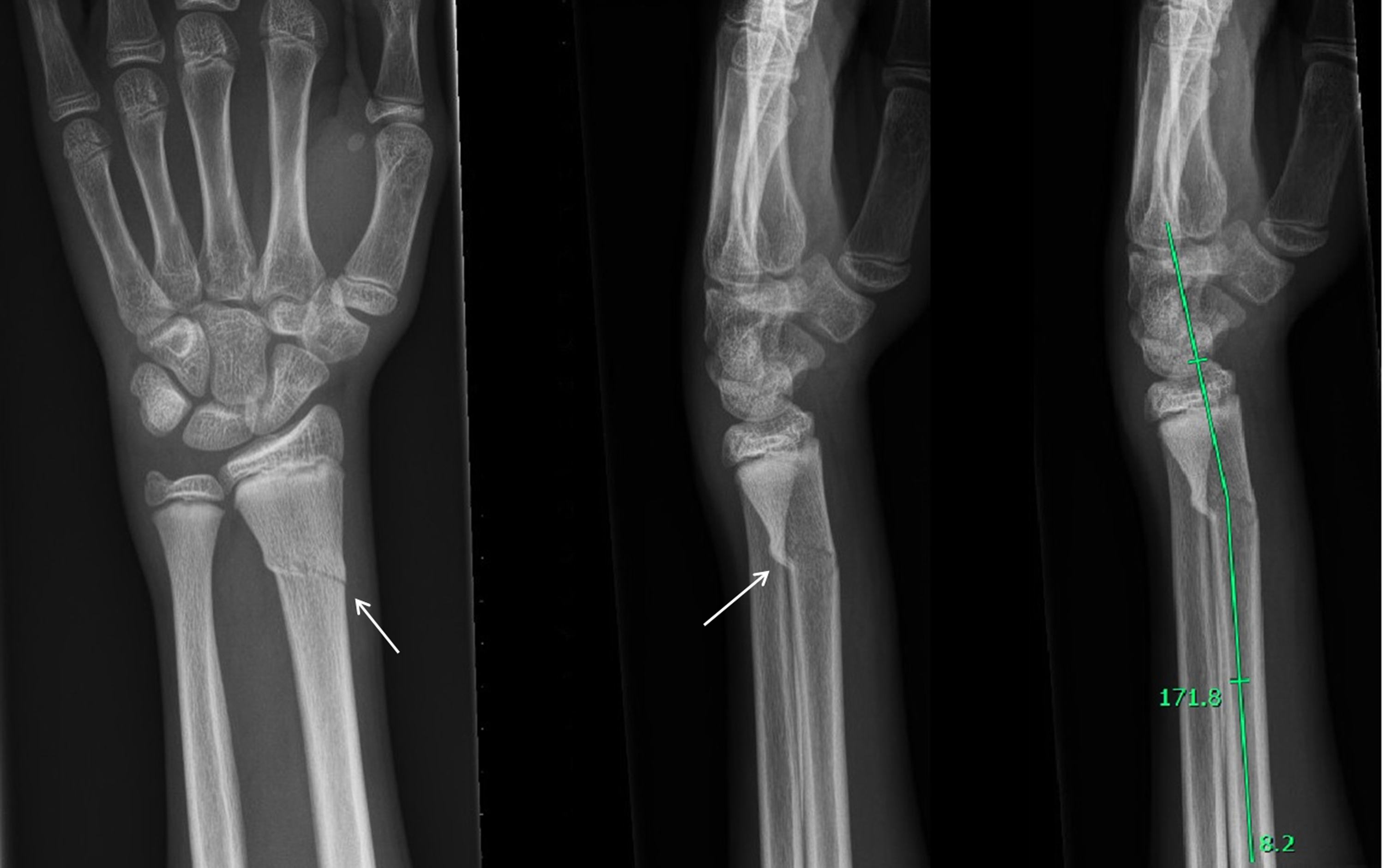

Supplement: Supplementary file 9 — A 13-year-old boy with a greenstick fracture with 8° of dorsal angulation, considered clinically relevant (PNG 1373 kb) [file 247_2018_4186_Fig9_ESM.png]

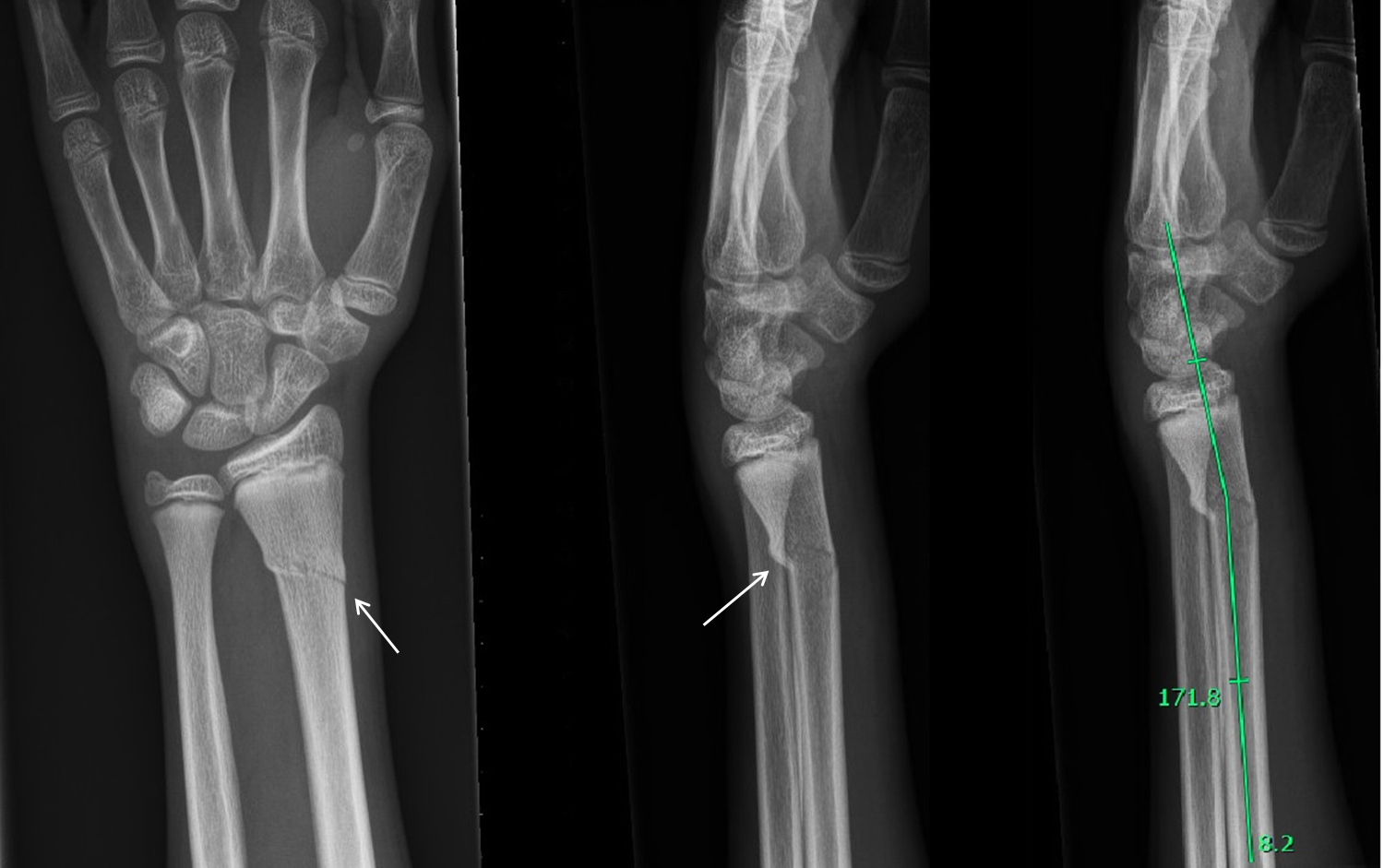

Supplement: Supplementary file 10 — High Resolution Image (TIF 1017 kb) [file 247_2018_4186_MOESM5_ESM.tif]
